# Solid pseudopapillary tumor of the pancreas and concomitant urogenital malformations in a young woman

**DOI:** 10.1186/1746-1596-8-35

**Published:** 2013-02-27

**Authors:** Zhi-Wei Guan, Lu Sun, Yan-Qiu Wang, Bai-Xuan Xu

**Affiliations:** 1 Department of Nuclear Medicine, Chinese PLA General Hospital, Ruxing road 28, Beijing 100853, China; 2 Department of Pathology, Chinese PLA General Hospital, Beijing, 100853, China; 3 Department of Ultrasound, Chinese PLA General Hospital, 28 Ruxing Road, Beijing, 100853, China

**Keywords:** Solid pseudopapillary tumor, Urogenital malformation, FDG PET/CT

## Abstract

**Abstract:**

Solid pseudopapillary tumor (SPT) of the pancreas is a rare pancreatic tumor with low malignant potential. It occurs characteristically more often in young women. SPT associated with extra- and pancreatic anomalies are occasionally reported. Here we report a case of pancreatic SPT with concomitant urogenital malformations including solitary kidney and uterus didelphys in a 25-year-old woman. The patient underwent central pancreatectomy, and SPT was confirmed with pathological results. Recurrence or metastasis was not found after 14 months of follow-up.

**Virtual Slides:**

The virtual slide(s) for this article can be found here: http://www.diagnosticpathology.diagnomx.eu/vs/4264758678755142

## Background

Solid pseudopapillary tumor (SPT) of the pancreas is rare, accounting for 0.13-2.7% of all pancreatic tumors [1]. It is unique, has low malignant potential and predominantly affects young women [1,2]. Radiological and pathological studies have revealed that the tumor is quite different from other pancreatic tumors [3-7]. But the cell origin of SPT and tumorigenesis are still enigmatic. Occasionally, SPT has been reported to be associated with extra- and pancreatic anomalies [8-11]. Here we present radiological, PET/CT and histological findings of a case of pancreatic SPT with concomitant urogenital malformations.

## Case presentation

**Figure 1:**
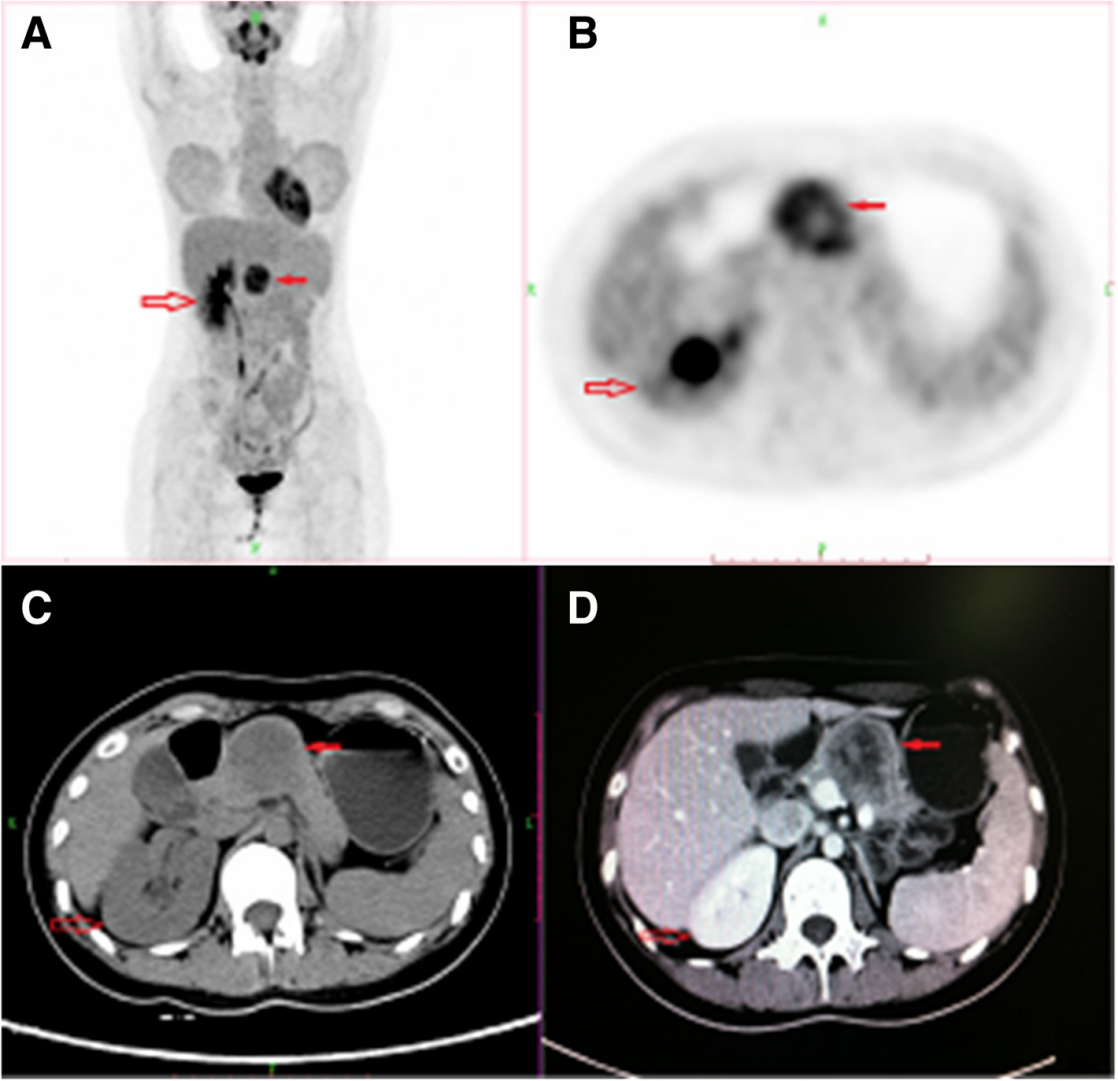
**The tumor was located in the body of the pancreas with intense FDG uptake (A-B, solid red arrow). **The tumor exhibited hypoattenuation on pre-contrast CT (**C**), and was enhanced inhomogeneously after contrast administration (**D**). The FDG accumulation was mainly located in the peripheral part of the tumor (**B**), corresponding to the solid part of the tumor with enhancement (**D**). Only the right kidney was found (**A-D**, red arrow).

**Figure 2:**
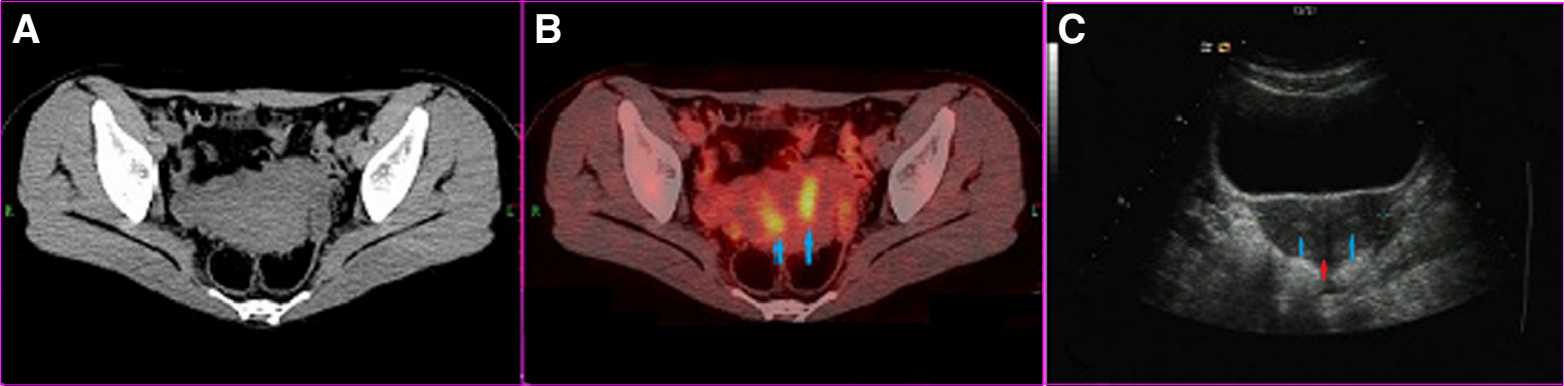
**Two linear foci of FDG accumulation in the central part of the uterus were found in the PET/CT fusion image (B, blue arrow). **Ultrasound scan showed two cavities (blue arrows) with muscular septum (red arrow) (**C**).

**Figure 3:**
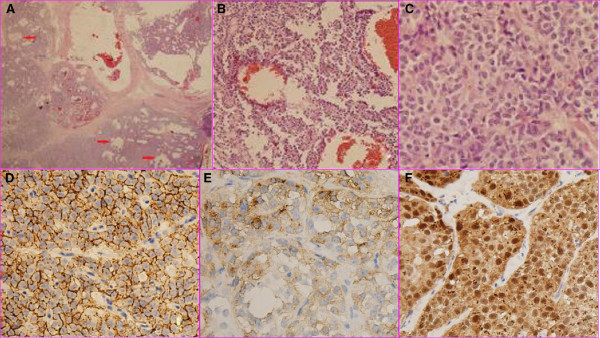
**A low power view showing the cystic components (arrowheads) of the tumor (H&E, magnification ×12.5, A). **Sheets and nests of uniform polygonal epithelioid cells with round or oval nuclei and acidophilic cytoplasm divided by thin fibrovascula stroma were found. Pseudopapillary structures were observed. Cell atypia was mild and mitosis was not found (H&E, magnification ×200, **B**; H&E, magnification ×400, **C**). Immunohistochemistry showed that the tumor cells were positive for CD56 (**D**), CD10 (**E**), and the nuclear type of β-catenin (**F**) (Magnification ×400, **D-F**).

A 25-year-old unmarried woman with no remarkable past or family history of neoplasms was admitted to our hospital due to a tumor located at the pancreatic neck, which was incidentally found by abdominal ultrasound during a medical check up. Physical examination revealed no abnormal findings. There were no abnormalities in clinical laboratory tests such as serum amylase level or tumor markers, including AFP, CA199, CEA, CA125, and CA724. Precontrast abdominal CT showed a round hypo-attenuation mass approximately 4.5 cm in diameter with clear margin. After contrast administration, the tumor displayed peripheral enhancement, corresponding to the solid part of the tumor. Hypo-attenuation without enhancement was depicted in the central zone corresponding to cystic degeneration. Pancreatic duct of the upper stream did not dilate. No parenchymal atrophy or vessel invasion was found. Accordingly, SPT was the most probable diagnosis, and nonfunctional PNET was considered as the differential diagnosis. Meanwhile, the left kidney was not found within the coverage of the abdominal CT scan. Later, the patient received FDG PET/CT scan covering from the skull base to the upper part of the thigh for further evaluation of the pancreatic tumor. With regard to the PET findings, the pancreatic tumor showed inhomogeneous FDG uptake (Figure 1) with a maximum standardized uptake value (SUVmax) of 7.5 in the peripheral zone of solid component. The left kidney was still not found. Interestingly, PET image showed two foci of slight FDG-accumulation at the uterine cavity (Figure 2B), and uterus didelphys were suspected. After consulting the clinical paper of the patient, we found that she had regular menses. She received abdominal ultrasound, and uterus didelphys was confirmed (Figure 2C). For this patient, PET/CT helped to diagnose the unexpected urogenital agesi, and to determine the stage of the pancreatic tumor as being confined without metastasis. After PET/CT scan, the patient underwent central pancreatectomy. Gross surgical specimen showed that the tumor was well encapsulated by a fibrous pseudocapsule with solid and cystic components. Microscopically, the tumor was composed of sheets and nests of uniform polygonal epithelioid cells with round or oval nuclei and acidophilic cytoplasm divided by thin fibrovascula stroma. Degenerative changes were seen in some parts of the tumor. Invasion of the vascular space or perineural invasion was not identified. The proliferation marker Ki-67 index was less than 5% and cellular atypia was mild. According to immunohistochemical analysis, the tumor cells showed nuclear type of β-catenin (++), CD10 (+), CD56 (+), Vimentin (+), and α1-antitrypsin (+), but were negative for chromogranin A (CgA) or Synaptophysin (Syn). These finding supported a final diagnosis of SPT of the pancreas (Figure 3). The patient had a smooth recovery and was released 10 days after resection. Recurrence or metastasis was not found after 14 months of follow-up.

## Discussion

SPT is rare and far less common than the other pancreatic tumors, including ductal adenocarcinoma, cystic and neuroendocrine tumors. It is occasionally found because of the vague symptoms, like those of some nonfunctional neuroendocrine tumors [12]. In our case, the pancreatic tumor was an occasional finding during physical check-up, and was typical in terms of demographic figure, radiological feature and pathological findings. First, it occurred in a young woman, which represented the typical population. Previous studies have shown that SPT predominantly affects young women from 20 to 40 years old [1]. Second, it exhibited the typical radiological features on CT, including regular shape, well-defined margin and inhomogeneous appearance corresponding to the solid and cystic texture [2,3,5,13]. Such radiological features are very different from those of ductal adenocarnoma, as the latter is always infiltrative with poorly defined margin and is invasive to the surrounding tissues. Last, the pathologic examination, light microscopy and immunohistological staining results of our case were typical. The tumor is characterized with fibroscapsule surrounding and varied protion of solid and cystic components showing hemorrhagic changes [1-3]. Under light microscope, the pseudopapillary structures and degenerative changes, such as necrosis, hemorrhage, cholesterol clefts and foamy macrophages, are characteristic findings [2]. Besides, the tumor cells are characteristically uniform with mild atypia and rare mitosis, indicating its benign entities [1,2]. It is relatively easy to differentiate SPT from ductal adenocarcinoma based on these histological features, as the latter is ofter more moderately or poorly differentiated and is composed of haphazardly arranged glands admixed with a dense desmoplastic stroma [14]. Cystic neoplasms, such as mucinous or serous cystic neoplasm, can also be easily differentiated from SPT, because they lack communication with the pancreatic duct system and have no mucinous or serous epithelium usually supported by an “ovarian” stroma [15,16]. Neuroendocrine tumors, especially the well-differentiated ones, are the most important entities in the differential diagnosis of SPT, because they may display similar light microscopic features, and neuroendocrine markers are variably expressed in SPT [1,2]. Excep for the consistently negative results for chromogranin A, expressions of other neuroendocrine markers such as synaptophysin, neuron-specific enolase and CD56 at various levels were demonstrated [1,2,17,18]. Recently, the nuclear type of β-catenin has been regarded as an unique immunohistochemical feature of SPT as it underlies the genetic mutation of catenin found in more than 90% of SPT [6,7]. Abnormal nuclear labeling of β-catenin strongly supports the diagnosis of SPT. SPT is largely benign according to its pathological features, but interestingly, it always shows hyper-metabolism of FDG on PET [19-21], which mimics malignant tumor. The high cellular density, rich mitochondria and the hypervascular nature as shown in radiological findings have been thought to contribute to the FDG accumulation [19]. In rare cases, SPT metastasizes to liver or transplants on peritomeum. In this patient, PET/CT staged the tumor to be confined without metastasis or transplantation, and she had safely undergone central pancreatectomy. Because of the indolent entity, patients usually have very good prognosis with less likelihood of recurrence and metastasis after resection of SPT [1,2]. Different from SPT, ductal adenocarcinoma always has very poor prognosis even after complete resection, as metastasis and recurrence are common.

In our case, the urogenital anomalies of solitary kidney and uterus didelphys were incidental findings. In premenopausal women, uteral endometrium always display physiologic FDG uptake, and can be easily recognized with its characteristically linear shape and central location in the uterus [22]. But different from other patients, the patient in this case had two separate linear FDG accumulations at the central part of her uterus, indicating two locations of endometrium. So uterus didelphys was suspected when we read the PET/CT image and was confirmed by ultrasound.

SPT has been reported to occur occasionally in patients with pancreatic anormalies such as pancreatic dorsal agenesis [9,11] and pancreatic divicum [10,23]. There is another report of SPT in a young woman with Mulvihill-Smith syndrome (MSS), a congenital mental anomaly characterized by progeria-like aspect, multiple pigmented nevi, mental retardation, microcephaly, low stature and lack of facial subcutaneous fat [8]. But this is the first case of SPT with concomitant urogenital anomalies. The findings are very interesting because both SPT and urogenital anomaly are very rare diseases. When they were found in one patient, we wondered whether the concomitant occurrence was just a coincidence.

Recently, β-catenin and Wnt signaling pathway has been found to play an important role in SPT tumorigenesis [7,24-26]. SPT almost consistently harbors β-catenin gene (CTNNB1) mutations in exon 3 [27], resulting in the activation of the Wnt-signaling pathway. The CTNNB1 gene mutations inactivate one of the glycogen synthase kinase-3β phosphorylation sites on the β-catenin protein, and block the degradation of β-catenin. The β-catenin protein binds to the T-cell transcription factor (Tcf)/lymphoidenhancer-bind-factor (Lef), and is then translocated to the nucleus, as indicated by nuclear expression of β-catenin [25,26,28]. In the nucleus, the β-catenin-Tcf/Lef complex activates the transcription of several oncogenic genes, including c-myc and cyclin D1. As a matter of fact, activation of β-catenin in mice induces large pancreatic tumor resembling human SPT, presenting *in vivo* evidence that β-catenin/Wnt signaling pathway plays a critical role in SPT tumorigenesis [29]. Renal agenesis (RA) is embryologically associated with genital malformations because Wolffian or mesonephric duct has inductive function on the Müllerian duct during normal development [30,31]. Patients with urogenital malformations always harbor Wnt muations or abnormal catenin [32,33]. Besides, urogenital malformations are induced in mice model by variable interruptions of the β-Catenin/Wnt-signaling pathway [34], but the exact molecular mechanisms are so complicated and far from complete disclosure.

As mentioned above, β-catenin and the Wnt-signaling pathway play important roles in both SPT tumorigenesis and urogenital malformations. We wondered whether the concurrent tumor of SPT and urogenital malformations in this young woman were triggered by a primary insult, which subsequently served to propagate aberrant urogenital development as well as SPT tumoregenesis, a process in which β-Catenin/Wnt-signaling pathway played important roles. But similar cases have not been found in the literature. The underlying molecular genetic mechanism and the relationship between SPT and the congenital anomalies need to be further investigated.

In conclusion, SPT is rare and specific in demography, radiology and pathology. It is benign and the prognosis is good after resection. It occasionally occurs in patients with extra- and pancreatic anomalies. The concurrent SPT and congenital malformations might be associated with the β-catenin/Wnt-signaling pathway. But the association needs to be confirmed.

### Consent

Written informed consent was obtained from the patient for publication of this case report and any accompanying images. A copy of the written consent is available for review by the Editor-in-Chief.

## Abbreviations

SPT: Solid pseudopapillary tumor; PET/CT: Positron emission tomography/computed tomography; FDG: Fluoro-deoxy-glucose.

## Competing interests

The authors declare no competing interests.

## Authors’ contributions

ZG contributed to PET/CT interpretation and literature review. ZG wrote the manuscript. LS performed histologic and immunohistochemic analyses. YW contributed to ultrasound interpretation. BX reviewed the manuscript and proof read the final version. All authors read and approved the final manuscript.
